# SULFATION PATHWAYS: A role for steroid sulphatase in intracrine regulation of endometrial decidualisation

**DOI:** 10.1530/JME-18-0037

**Published:** 2018-05-02

**Authors:** Douglas A Gibson, Paul A Foster, Ioannis Simitsidellis, Hilary O D Critchley, Olympia Kelepouri, Frances Collins, Philippa T K Saunders

**Affiliations:** 1 MRC Centre for Inflammation ResearchThe University of Edinburgh, QMRI, Edinburgh, UK; 2 Institute of Metabolism & Systems ResearchUniversity of Birmingham, Birmingham, UK.; 3 MRC Centre for Reproductive HealthThe University of Edinburgh, QMRI, Edinburgh, UK

**Keywords:** decidualisation, steroid sulphatase, sulphation, Irosustat, estrone

## Abstract

In women, establishment of pregnancy is dependent upon ‘fine-tuning’ of the endometrial microenvironment, which is mediated by terminal differentiation (decidualisation) of endometrial stromal fibroblasts (ESFs). We have demonstrated that intracrine steroid metabolism plays a key role in regulating decidualisation and is essential for time-dependent expression of key factors required for endometrial receptivity. The primary aim of the current study was to determine whether sulphated steroids can act as precursors to bioactive sex steroids during decidualisation. We used primary human ESF and a robust *in vitro* model of decidualisation to assess the expression of genes associated with sulphation, desulphation and transport of sulphated steroids in human ESF as well as the impact of the steroid sulphatase (STS) inhibitor STX64 (Irosustat). We found evidence for an increase in both expression and activity of STS in response to a decidualisation stimulus with abrogation of oestrone biosynthesis and decreased secretion of the decidualisation marker IGFBP1 in the presence of STX64. These results provide novel insight into the contribution of STS to the intracrine regulation of decidualisation.

## Introduction

Decidualisation is a fundamental process of endometrial remodelling that is required for the establishment of pregnancy. It is associated with unique time-dependent transcriptomic and proteomic changes (reviewed in Gellersen & Brosens 2014), which are reported to be disordered in women with recurrent implantation failure (Ruiz-Alonso *et al*. 2013, Koot *et al*. 2016). Whilst the post-ovulatory rise in progesterone acts as an *endocrine* signal to stimulate decidualisation of oestrogen-primed human endometrial stromal fibroblast (hESF) (reviewed in Gellersen & Brosens 2014), we have demonstrated an important role for *local* (intracrine) steroid metabolism in fine-tuning the cellular differentiation of hESFs (Gibson *et al*. 2016*b*). Importantly, we have established that expression of *CYP19A1* (aromatase, the key enzyme required for conversion of androgens to estrogens) as well as *AKR1C3* and *SRD5A1* (enzymes that convert precursor androgens into testosterone and dihydrotestosterone (DHT), respectively) are altered in a time-dependent manner (Gibson *et al*. 2013, 2016*a*). Expression of these enzymes results in increased biosynthesis of potent steroid receptor agonists (E2, testosterone, DHT) that in turn regulate the expression of genes important for receptivity and immune-cell-mediated vascular remodelling (Gibson *et al*. 2013, 2015, 2016*a*).

Bioavailability of sulphated steroids can affect intra-tissue concentrations of oestrogens and androgens, which may play a major role in regulating the function of both normal and malignant tissues (Mueller *et al*. 2015). Hydrolysis of sulphated precursors into biologically active steroids requires the enzyme steroid sulphatase (STS) (Fournier & Poirier 2009). STS primarily converts oestrone sulphate (E_1_S) to oestrone (E1) and is expressed in many tissues, albeit at low levels (Miki *et al*. 2002, Suzuki *et al*. 2003). In addition to oestrogen-forming activities, STS also has the capacity to hydrolyse DHEAS to DHEA, which may also act as a precursor for the formation of oestrogens and androgens within target tissues (Purohit *et al*. 2011). The actions of STS are countered by sulphotransferases that promote inactivation and metabolism of steroids following conjugation of a sulphate moiety. Sulphotransferases are a diverse gene family and the isozymes SULT1A1, SULT1E1, SULT2A1 and SULT2B1 have been associated with sulphation of steroids (Mueller *et al*. 2015). SULT1E1 primarily catalyses the sulphation of oestrogens, although SULT1A1 is also reported to have this action (Gamage *et al*. 2005). Sulphation requires the co-substrate PAPS (adenosine 3′-phosphate 5′-phosphosulfate) that provides the universal sulphate donor compound for all sulphotransferase reactions. PAPS is synthesised via one of two PAPS synthase isoforms PAPSS1 and PAPSS2 (Schroder *et al*. 2012). Uptake and excretion of sulphated steroids from cells is regulated by membrane transporters which are reported to be expressed in endometrial and ovarian cancer cells and tissues (Mueller *et al*. 2015, Rizner *et al*. 2017). To date, few studies have examined the expression of STS, sulphotransferases, PAPSS or membrane transporters in endometrial tissues. 

STS immunoexpression has been detected in both stromal and epithelial cells in the endometrium across the menstrual cycle (Dassen *et al*. 2007). In a study using 12 endometrial cancer samples and adjacent endometrium, expression of both *STS* and *SULT1E1* mRNAs were higher in the normal endometrium than in the malignant cells (Smuc *et al*. 2006). Notably, in both benign and normal endometrial tissues, concentrations of mRNAs for *STS* were higher than *SULT1E1* (Smuc *et al*. 2006), which may favour an increase in the bioavailability of active steroid receptor agonists in the endometrium. Indeed, studies using STX64 (Irosustat), an irreversible inhibitor of STS (Day *et al*. 2009), suggest that STS can regulate formation of oestradiol (E2) in the uterus (Colette *et al*. 2011). STS inhibitors are also reported to decrease the oestrogenic growth of hormone-dependent tissues, including endometrial cancer tissues (Foster *et al*. 2008) and proliferation of human endometrial xenografts (Colette *et al*. 2011). Recently, Sinreih *et al.* extended their studies on endometrial cancer steroid intracrinology and concluded that E2 was formed from E_1_S via the sulphatase pathway rather than via aromatase in endometrial cancer tissues (Sinreih *et al*. 2017). In contrast, previous studies in both human and mouse suggest intracrine regulation of the normal endometrium occurs via aromatase-dependent biosynthesis of oestrogens (Das *et al*. 2009, 2012, Gibson *et al*. 2013).

In the current study, we sought to complement and extend our previous investigations by assessing the contribution of STS to intracrine regulation of decidualisation. We used an *in vitro* model of decidualisation to assess the expression of genes associated with sulphation, desulphation and transport of sulphated steroids in hESFs and assessed the impact of the STS inhibitor STX64 (Irosustat). Our results provide new insight into the mechanisms that contribute to generation of an oestrogen-rich microenvironment during the establishment of pregnancy.

## Materials and methods

### Human studies

Primary human endometrial tissue (proliferative phase, *n* = 9) was obtained from women undergoing surgery for non-malignant gynaecological conditions. None of the women were receiving hormonal therapy or suffering from endometriosis. Primary hESFs were isolated from proliferative phase endometrium and cycle phase determined as reported previously (Bombail *et al*. 2010). Briefly, endometrial tissue was minced using scalpel blades, followed by DNAse/collagenase digest for 2 h at 37°C. The tissue homogenate was then sequentially strained through 70 µm and 40 µm membrane filters to separate hESF from the glandular epithelium. Isolated hESFs were washed in warmed PBS and cultured in RPMI 1640, supplemented with 10% foetal calf serum (FCS) at 37°C in 5% CO_2_ and maintained for a maximum of five passages. Forty-eight hours prior to experimentation, hESF culture media were changed to phenol red-free RPMI 1640 supplemented with 10% charcoal-stripped FCS (CSFCS). Decidualisation was induced by addition of decidualisation (DEC) media (phenol red-free RPMI 1640, 2% CSFCS, 0.1 mg/mL 8-Br-cAMP (Sigma, B5386), 1 µM progesterone (Tocris, Abingdon, UK; Cat no. 2835)). Some cell cultures were supplemented with the STS inhibitor Irosustat (STX64; 10 µM, Sigma S1950) for the duration of the culture period. Control cultures were incubated with phenol red-free RPMI 1640, 2% CSFCS and equivalent volume of vehicle control (DMSO). 2 × 10^5^ hESFs were seeded per well of six-well plate, treatments were in duplicate and assessed in minimum of three individual patients for all treatments. For experiments assessing expression and activity of sulphation enzymes/transporters, cells from *n* = 6 patients were assessed. For experiments assessing the impact of STX64, cells from *n* = 3 patients were used. Each sample was assayed in duplicate and average values were analysed. To assess the time-dependent accumulation of secreted products, treatments were maintained for the duration of each time point. hESFs were treated for 24 h (1 day), 48 h (2 days), 4 days or 8 days as indicated; cells were treated in parallel with dedicated samples for each time point and patient. The expression of genes associated with sulphation, desulphation and transport of sulphated steroids was assessed by RT-qPCR.

### Assessment of mRNA

Isolation of mRNAs, preparation of cDNAs and analysis by RT-qPCR was performed according to standard protocols (Bombail *et al*. 2010); samples were analysed by the comparative ΔΔCt method with *CYC* (cyclophilin) as an internal control. Primers/probes are given in Supplementary Table 1 (see section on supplementary data given at the end of this article).

### ELISA

Insulin-like growth factor-binding protein 1 (IGFBP1), E1 and E2 in culture supernatants were determined by ELISA as described previously (Gibson *et al*. 2013, 2016*a*). E1 and E2 were not detected in control supernatants (not shown). Antibody cross-reactivity for E1 and E2 ELISA was <5% for other steroids (Supplementary Tables 2 and 3).

### Measurement of steroid sulfatase activity

STS activity was determined in cell lysates as described previously (Purohit *et al*. 1997). Briefly, cells were lysed in RIPA buffer and protein concentration measured by BCA assay. Hundred micrograms of protein were incubated for 4 h with PBS containing [6,7-^3^H] E_1_S (4 × 10^5^ dpm) adjusted to a final concentration of 20 μM with unlabelled E_1_S. [4-^14^C] E1 (1 × 10^4^ dpm) was used to monitor procedural losses. E1 was separated from E_1_S by toluene partition and ^3^H and ^14^C radioactivity was measured by liquid scintillation spectrometry. Results were expressed as E1 formed pmol/h/mg protein.

### Statistics

Statistical analysis was performed using GraphPad prism. Two-way ANOVA was used to determine the significance between treatments in grouped data; interaction between time and treatment was assessed, Sidak’s multiple comparisons test was used to assess the differences between treatments for individual time points. Non-parametric testing was utilised where sample sizes were insufficient to confirm normality of data distribution; Mann–Whitney test was used to assess differences between treatments at each time point for RT-qPCR data. Criterion for significance was *P* < 0.05. All data are presented as mean ± s.e.m.

### Study approval

Written informed consent was obtained from all subjects prior to surgery; ethical approval was granted by the Lothian Research Ethics Committee (LREC/07/S1103/29 and LREC 10/S1402/59). Methods were carried out in accordance with NHS Lothian Tissue Governance guidelines. Studies using these cells have previously been reported in (Gibson *et al*. 2018).

## Results

### Expression of sulphation and desulphation enzymes during decidualisation of human ESF

**Figure 1:**
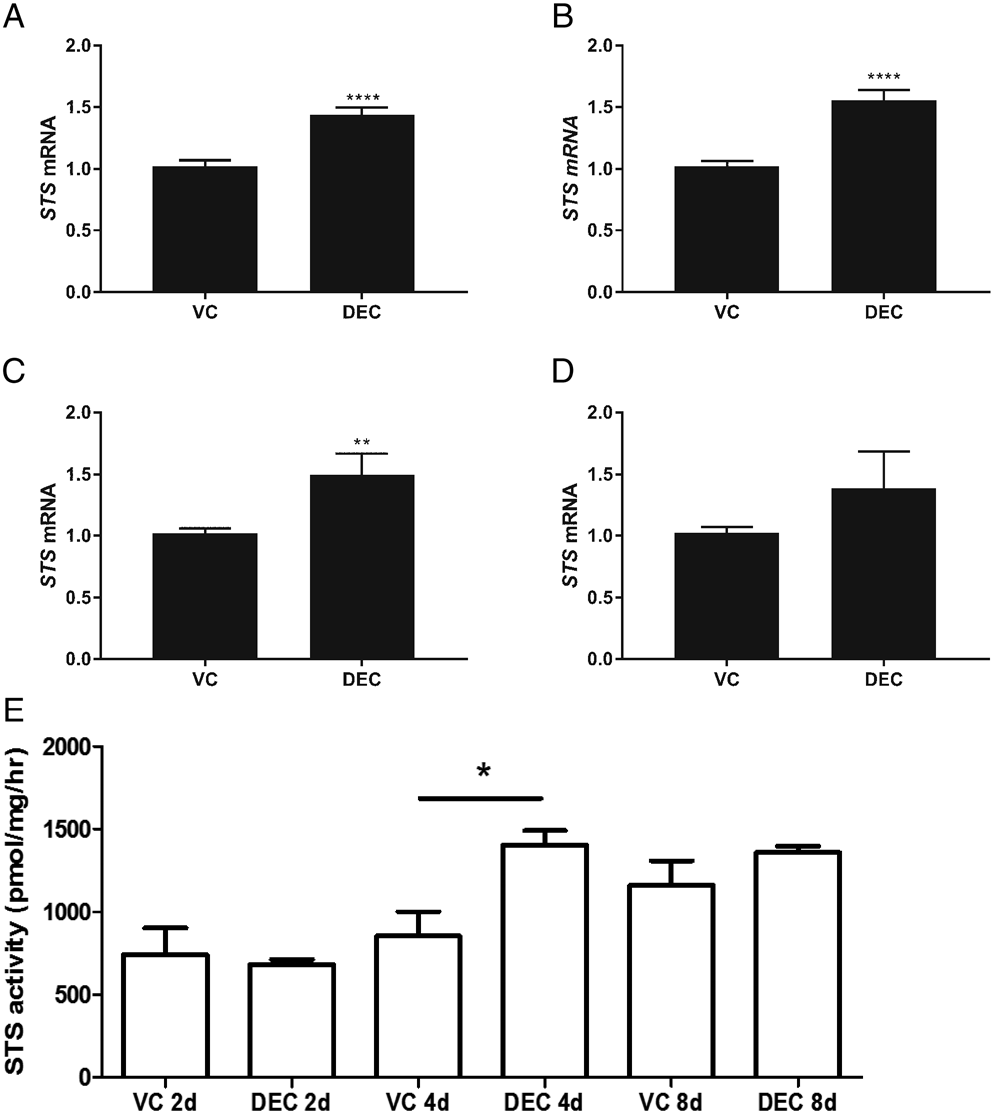
Expression and activity of STS during decidualisation. HESFs were treated with either VC or DEC for 1–8 days as indicated and mRNAs encoding *STS* were assessed by RT-qPCR. *STS* mRNA was significantly increased in hESF treated with decidualisation media for 1, 2 and 4 days compared to VC but not different at the 8-day time point (A, B, C and D; *N* = 6 patients, duplicate treatments; Mann–Whitney test.). STS activity was assessed in hESF (E) and was increased in cells treated with DEC compared to VC after 4 days (*P* < 0.05; *n* = 6 patients, one-way ANOVA). *P < 0.05; **P < 0.01; ****P < 0.0001. DEC, decidualisation media; HESF, human endometrial stromal fibroblasts; STS, steroid sulphatase; VC, vehicle control.

**Figure 2:**
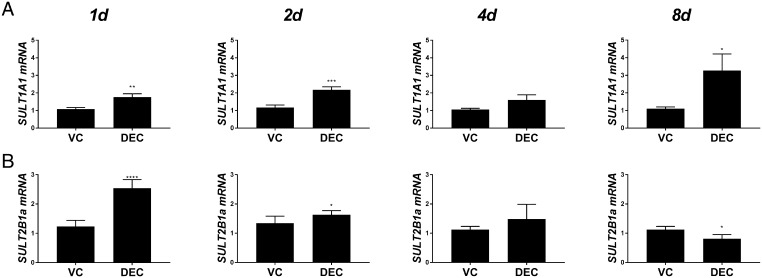
Expression of sulphation enzymes during decidualisation. HESFs were treated with either VC or DEC for 1–8 days as indicated and mRNAs encoding *SULT2B1 transcript variant 1* (*SULT2B1a*) and *SULT1A1* were assessed by RT-qPCR. (A) *SULT1A1* mRNA was significantly increased at 1, 2 and 8 days in hESF treated with DEC compared to VC. (B) *SULT2B1a* mRNA was significantly increased after 1- and 2-day treatment with DEC but by 8 days was significantly decreased compared to VC treatment. Expression of *SULT1E1* and *SULT2A1* was not detected (not shown). *n* = 6 patients, duplicate treatments. Mann–Whitney test. *P < 0.05; **P < 0.01; ***P < 0.001; ****P < 0.0001. DEC, decidualisation media; HESF, human endometrial stromal fibroblasts; VC, vehicle control.

We assessed expression of *STS* as well as members of the sulphotransferase gene family in cells recovered 1–8 days after incubation with control (VC) or DEC culture media (Figs 1 and 2). Expression of STS was increased at all time points in hESF stimulated with DEC; results reached statistical significance in samples recovered on Day 1 (Fig. 1A; *n* = 6; *P* < 0.0001), 2 (Fig. 1B; *n* = 6; *P* < 0.0001) and 4 (Fig. 1C; *n* = 6; *P* < 0.01). To complement these findings, STS activity was assessed in cell lysates and was significantly increased in lysates from hESF treated with DEC media compared to VC after 4 days of treatment (Fig. 1E, *n* = 6; *P* < 0.05).

To determine whether E1 and E2 formed by decidual cells (Gibson *et al*. 2013) might also be subject to inactivation, we also assessed expression of sulphotransferases in the same samples. In these experiments, expression of *SULT1E1* and *SULT2A1* mRNAs were not detected (not shown). In contrast, mRNAs encoded by *SULT1A1* and *SULT2B1a* were present (Fig. 2A and B). *SULT1A1* mRNAs were significantly increased in hESF stimulated with DEC media compared to VC after 1 (*n* = 6; *P* < 0.01), 2 (*n* = 6; *P* < 0.001) and 8 days (*n* = 6; *P* < 0.05) of culture. *SULT2B1a* mRNAs were significantly increased on days 1 (*n* = 6; *P* < 0.0001) and 2 (*n* = 6; *P* < 0.01) of culture, however, by day 8, this was reversed with a significant decrease detected in DEC compared to VC-treated hESF (*n* = 6; *P* < 0.05).

### Expression of PAPSS isozymes during decidualisation of hESF

**Figure 3:**
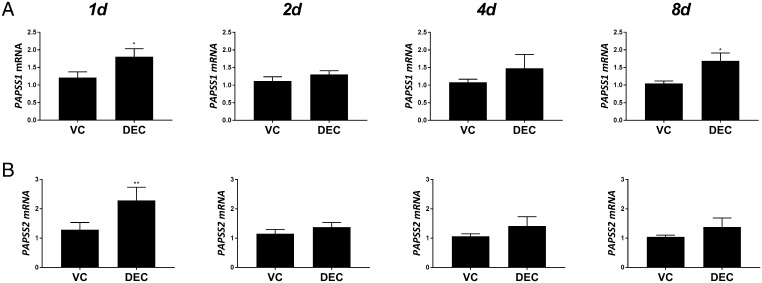
Expression of PAPSS isozymes during decidualisation. HESF were treated with either VC or DEC for 1–8 days as indicated and mRNAs encoding *PAPSS1* and *PAPSS2* were assessed by RT-qPCR. (A) *PAPPS1* mRNA was significantly increased at 1 and 8 days in hESF treated with DEC compared to VC while *PAPSS2* mRNA (B) was increased only after 1 day. *n* = 6 patients, duplicate treatments. Mann-Whitney test. *P < 0.05; **P < 0.01. DEC, decidualisation media; HESF, human endometrial stromal fibroblasts; PAPSS, adenosine 3′-phosphate 5′-phosphosulfate synthase; VC, vehicle control.

To complement and extend the studies on sulphotransferase enzymes, we also assessed expression of PAPSS isozymes *PAPSS1* and *PAPSS2*. Expression of mRNAs encoded by *PAPSS1* (Fig. 3A) was increased in DEC-treated hESF compared to controls on days 1 (*n* = 6; *P* < 0.05) and 8 (*n* = 6; *P* < 0.01), whereas PAPSS2 mRNA concentrations were only higher on day 1 (*n* = 6; *P* < 0.01) and unchanged at other time points (Fig. 3B).

### Expression of ATP-binding cassette transporters during decidualisation of human ESF

**Figure 4:**
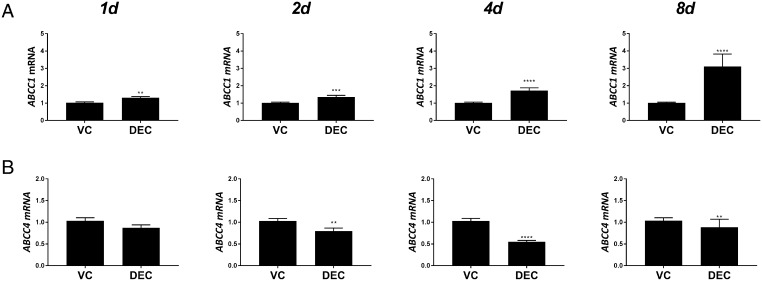
Expression of ATP-binding cassette transporters during decidualisation. HESFs were treated with either VC or DEC for 1–8 days as indicated and mRNAs encoding *ABCC1* and ABCC4 were assessed by RT-qPCR. (A) *ABCC1* mRNA increased in a time-dependent manner and was significantly increased in hESF treated with decidualisation media for 1, 2, 4 and 8 days compared to VC. (B) *ABCC4* mRNA decreased in a time-dependent manner and was significantly decreased after 2, 4 and 8 days of treatment with DEC compared to VC. Expression of mRNAs encoding the organic anion transporters *SLCO1A2*, *SLCO1B1*, *SLCO1B3* and *SLCO2B1 SULT1E1* were not detected. *n* = 6 patients, duplicate treatments. Mann–Whitney test. **P < 0.01; ***P < 0.001; ****P < 0.0001. DEC, decidualisation media; HESF, human endometrial stromal fibroblasts; VC, vehicle control.

We assessed expression of cellular transporters ABCC1 and ABCC4, which are associated with efflux of sulphated steroids. Expression of mRNAs encoding *ABCC1* was increased in a time-dependent manner in decidualised hESF (Fig. 4A). *ABCC1* mRNA expression was significantly increased in DEC compared to controls at each time point; 1 (*n* = 6; *P* < 0.0001), 2 (*n* = 6; *P* < 0.0001) and 4 (*n* = 6; *P* < 0.01) days, with the greatest increase in expression detected after 8 days (*n* = 6; *P* < 0.0001). In contrast, *ABCC4* mRNA expression was significantly decreased in hESF treated with DEC compared to controls at 2 (*n* = 6; *P* < 0.01), 4 (*n* = 6; *P* < 0.0001) and 8 (*n* = 6; *P* < 0.01) days (Fig. 4B). Expression of organic anion-transporting polypeptide transporters (SLC gene family; *SLCO1A2*, *SLCO1B1*, *SLCO1B3* and *SLCO2B1*), which mediate influx of sulphated steroids were not detected in either control or decidualised hESF (not shown).

In light of these results and as a complement to our previous studies in which we demonstrated that both E1 and E2 are secreted into culture media during our decidualisation protocol (as determined by ELISA (Gibson *et al*. 2013)), we used sensitive LC-MS to assess the concentrations of E_1_S or E_2_S in conditioned media from hESF, however, neither steroid was detected in either the control or DEC samples (not shown; LLOD for E_1_S and E_2_S = 0.5 ng/mL).

### Irosustat (STX64) disrupts bioavailability of oestrogens and alters decidualisation responses

**Figure 5:**
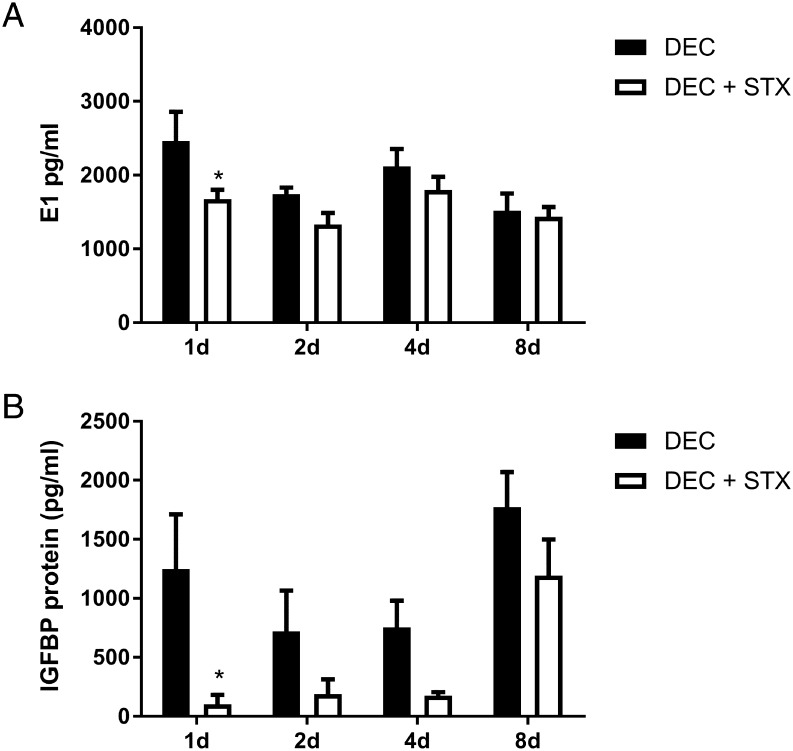
Irosustat (STX64) disrupts oestrogen bioavailability and alters decidualisation responses. The impact of the potent irreversible STS inhibitor STX64 (STX) on decidualisation of hESF was investigated following treatment for 1–8 days as indicated. (A) Secretion of E1 was significantly decreased after 1 day in hESF treated with DEC + STX compared to DEC alone (*P* < 0.05). Two-way ANOVA; global analysis time factor *P* = 0.0158, treatment factor *P* = 0.0111. Sidak’s multiple comparisons test; treatment effect significantly different day 1 *P* < 0.05. (B) Concentrations of IGFBP1 tended to be lower at all time points in hESF treated with DEC + STX compared to DEC alone. STX significantly decreased secretion of the decidualisation marker IGFBP1 after 1 day of treatment (*P* < 0.05). Two-way ANOVA; global analysis time factor *P* = 0.0026, treatment factor *P* = 0.0008. Sidak’s multiple comparisons test; treatment effect significantly different day 1 *P* < 0.05. E1 and IGFBP1 secretion was determined by ELISA. *n* = 3 patients, duplicate treatments. *P < 0.05. DEC, decidualisation media; E1, oestrone; HESF, human endometrial stromal fibroblasts; IGFBP1, insulin-like growth factor-binding protein-1.

We investigated the potential importance of STS activity during decidualisation of hESF by assessing the impact of the selective, irreversible STS inhibitor Irosustat (STX64). hESFs were stimulated with DEC media or co-incubated with STX64 (DEC STX) for 1, 2, 4 or 8 days. Incubation with STX64 was associated with altered production of oestrogens from hESF and mean concentrations of secreted E1 were decreased at each time point in hESF treated with DEC STX compared to DEC alone (Fig. 5A). The impact of STX on E1 secretion was time-dependent with the greatest reduction detected after 1 day (32% decrease in E1; *n* = 3 patients, *P* < 0.05) but at later time points, there was no significant difference between hESF treated with DEC and DEC STX. In contrast to E1, concentrations of secreted E2 were not altered between hESF treated with either DEC alone or DEC STX (Supplementary Fig. 1). We next assessed if altered bioavailability of oestrogens affected decidualisation of hESF. Notably, STX64 altered secretion of the decidualisation marker IGFBP1; with concentrations reduced at each time point and significantly decreased after 1 day in hESF treated with DEC STX compared to DEC alone (Fig. 5B, *n* = 3 patients, *P* < 0.05).

## Discussion

Oestrogens within tissues can be derived from two pathways; *de novo* synthesis, which requires aromatase, and activation of circulating sulphated steroids which requires STS (Simpson *et al*. 1994, Mueller *et al*. 2015). While *de novo* oestrogen synthesis via aromatase has emerged as a key steroidogenic process in the normal endometrium (Das *et al*. 2009, 2012, Gibson *et al*. 2013), activation of sulphated steroids via STS is reported to be a prominent mechanism of oestrogen activation in pathologies of the endometrium including endometrial cancer and endometriosis (Colette *et al*. 2011, Sinreih *et al*. 2017). To date, the literature supports that aromatase-dependent *de novo* synthesis of oestrogens is essential for intracrine regulation of endometrial function. Das *et al.* provided the first evidence that *de novo* synthesis of oestrogens was required for decidualisation (Das *et al*. 2009). Using a mouse model, they showed that following ovariectomy on day 5 of pregnancy, supplementation with progesterone was sufficient to maintain the decidualisation response as well as growth and development of implanted embryos, (Das *et al*. 2009). However, in the presence of the aromatase inhibitor letrozole, both decidualisation and implantation were impaired indicating that ovarian oestrogens were dispensable for decidualisation (Das *et al*. 2009). In further experiments using a mouse model of artificially induced decidualisation, they went on to show that factors required for stromal remodelling and neovascularisation were dependent on aromatase-dependent intrauterine *de novo* biosynthesis of oestrogens (Das *et al*. 2009, 2012). Our recent studies have extended these observations in mice and found evidence for complementary mechanisms in human endometrial stromal fibroblasts. We have shown that hESF have the capacity to utilise DHEA as a substrate and that hESF synthesise aromatisable androgens during decidualisation (Gibson *et al*. 2013, 2016*a*, 2018). We have demonstrated that decidualisation is characterized by an increase in the expression and activity of aromatase (CYP19A1) leading to local biosynthesis of both E1 and E2 within the microenvironment of the endometrial stroma (Gibson *et al*. 2013). We have also demonstrated that oestrogens regulate cellular cross-talk within the endometrium during the establishment of pregnancy by directly regulating immune cell function and promoting vascular remodelling (Gibson *et al*. 2015). Given that oestrogens play such an essential role in modulating the endometrial environment, in the present study, we sought to further examine the mechanisms that regulate the availability of oestrogens by assessing the contribution of STS to intracrine oestrogen bioavailability during decidualisation.

We speculated that in addition to the classic *de novo* biosynthesis pathway, oestrogens may also be synthesised via de-sulphation of E_1_S. Using a robust model of *in vitro* decidualisation and hESF, we detected increased expression and activity of STS. Notably, we found that the STS inhibitor STX64 (Irosustat) had a time-dependent impact on decidualisation and decreased secretion of IGFBP1 from days 1 to 4 of the time course. Decreased secretion of IGFBP1 was associated with concomitant decreases in E1 concentrations suggesting STS-dependent regulation of intracrine oestrogens occurs during decidualisation. Notably, the aromatase inhibitor letrozole is reported to completely inhibit uterine oestrogen production in ovariectomised mice (Das *et al*. 2009). However, in our previous study, we co-incubated hESF with the aromatase inhibitor letrozole during decidualisation, and this only reduced secretion of E1 and E2 by approximately 65% (Gibson *et al*. 2013). In the current study, E1 synthesis was reduced by up to 30% during decidualisation consistent with both STS- and aromatase-dependent production of E1 during decidualisation. Notably, the effect of STX64 on E1 concentrations was most pronounced at day 1 but absent by day 8. This reflects our previous observations regarding the role of intracrine sex steroids during decidualisation, whereby modulation of local androgen action resulted in time-dependent changes in the expression of decidualisation and implantation-associated factors in the presence of the antiandrogen flutamide (Gibson *et al*. 2016*a*). Thus, if sulphated *oestrogens* are contributing to the ‘oestrogen pool’ during decidualisation, this effect may also be temporally regulated, with increased de-sulphation of steroid precursors during the early stages of decidualisation preceding aromatase-dependent synthesis of oestrogens during the latter stages of the differentiation process. Our results therefore suggest sulphation pathways contribute to regulation of decidualisation via rapid as well as gradual mechanisms. Taken together, our findings suggest bioavailability of intracrine oestrogens modulates initiation of stromal decidualisation and that tightly controlled regulation of intracrine steroids, via sulphation and export, is required as decidualisation progresses for appropriate paracrine signalling to immune and vascular endothelial cells within the tissue microenvironment during endometrial remodelling. 

Notably, the most abundant oestrogen detected in the current analysis and in our previous study (Gibson *et al*. 2013) was E1. This was surprising as E1 is classically considered to be weak/inactive oestrogen. However, our results are consistent with Huhtinen *et al.* who performed intra-tissue profiling of human endometrial tissues using LC-MS. They reported increased concentrations of E1 in secretory phase endometrium compared to serum concentrations and that mean intrauterine concentrations of E1 are greater than E2 in the secretory phase (Huhtinen *et al*. 2012). Although the role of E2 in regulating establishment of pregnancy is well established, the predominance of E1 in the intrauterine environment during the secretory phase may suggest an independent role for E1 in regulating endometrial function. We have previously demonstrated that equimolar concentrations of E1 (10 nM) can directly regulate increases in ER-dependent migration of uterine NK cells to a similar extent as E2 (Gibson *et al*. 2015) and in the current study, it was notable that reduction of E1 concentrations but not E2 by STX64 abrogated secretion of the decidualisation marker IGFBP1. Whether E1 acts as a direct agonist or by modulating intracrine metabolism in this context requires further investigation. However, the findings in the current study provide new evidence that availability of E1 may independently contribute to regulation of the endometrial microenvironment.

Sulphation of steroids is a primary route for metabolism of active oestrogens in healthy peripheral tissues. Previous studies have demonstrated that mRNA expression of the main oestrogen sulphating enzyme SULT1E1 is detected at all stages of the menstrual cycle in total endometrial tissue extracts (Dassen *et al*. 2007). In the current study, *SULT1E1* was not detected in hESF, consistent with classic metabolism studies, which only detected formation of E_1_S from isolated endometrial epithelial, but not stromal, cells following incubation with E2 (Liu & Tseng 1979). We did detect mRNA expression of *SULT1A1* which is reported to be expressed in the endometrium (Rubin *et al*. 1999) and also has oestrogen sulphating activity (Falany & Falany 1996), albeit in the micromolar range. Notably, increased concentrations of sulphotransferases (SULT1A1, SULT2B1) were detected concurrent with increased expression of PAPSS isozymes; however, we could not detect any sulphated oestrogens in conditioned media from hESF. There are limited data on the expression of transmembrane transporters that mediate cellular influx and efflux of sulphated steroids in the endometrium. Nishimura and Naito assessed mRNA expression of 46 ABC transporters and 108 SLC transporters in a range of tissues including 3 human uterine samples (Nishimura & Naito 2005). Notably, relative expression of mRNAs (ratio of target genes to *PPIA* housekeeping gene) encoding *SLCO1A2*, *SLCO1B1* and *SLCO2B1* as well as *ABCC1* and *ABCC4* were reported in uterine samples; although expression of SLC isoforms was relatively low compared to other tissues (Nishimura & Naito 2005). In the current study, we could not detect expression of SLC isoforms in hESF, however, ABC transporters (ABCC1 and ABCC4) were detected and dynamically regulated consistent with a possible role in export of sulphated steroids during decidualisation. It is a limitation of the current study that only the contribution of stromal compartment was assessed. Given the reported expression and activity of SULT1E1 in endometrial epithelial cells (Liu & Tseng 1979), further studies are needed to assess the contribution of epithelial cells and stromal–epithelial interactions in regulating oestrogen bioavailability within the endometrium. Our data support STS activity is prominent in the stromal compartment during decidualisation and additionally we found some evidence for time-dependent increases in expression of sulphotransferase enzymes. We did not detect any secretion of sulphated oestrogens in conditioned medium for hESF, which may suggest that any sulphated oestrogens formed during decidualisation are subject to direct hydrolysis by STS.

In our study, we used primary human cells and a well-characterised model of *in vitro* decidualisation; however, it is conceivable that adaptive metabolism and alternative pathways may be more prominent in disease states such as endometriosis and endometrial cancer where increased expression of STS has been reported (Utsunomiya *et al*. 2004, Piccinato *et al*. 2016, Sinreih *et al*. 2017). It is notable that balanced availability of oestrogens is required to regulate establishment of pregnancy. Increasing or decreasing E2 outside the normal physiological range is detrimental to implantation in mice (Ma *et al*. 2003). Furthermore, supraphysiological levels of E2 and progesterone as a result of controlled ovarian hyperstimulation during *in vitro* fertilization treatment protocols is associated with low implantation rates, possibly as a result of an altered steroid milieu (Pellicer *et al*. 1996). ‘Out of phase’ endometrial remodelling is a common feature of implantation failure but whether dysregulation of endometrial intracrine metabolism occurs in sub/infertility requires further investigation. Notably, drugs that target steroid metabolism such as aromatase inhibitors, STS inhibitors, DASI (dual aromatase–STS inhibitors) and 17BHSD1 inhibitors may affect endometrial function, and these potential actions should be considered in future therapeutic applications of these drugs in reproductive-aged women.

## Conclusions

In the current study, we assessed the expression of sulphation and desulphation enzymes, sulphate donors and transmembrane transporters in hESFs during a time course of *in vitro* decidualisation. We found that inhibition of STS activity disrupted bioavailability of oestrogens and inhibited decidualisation responses consistent with stromal utilisation of sulphated steroids as precursors to active hormones during decidualisation. Elucidation of the complex intracrine metabolism of steroids within the endometrium during decidualisation will be critical to understanding the relevance of these findings to reproductive health and disease. The results of the current study provide new insight into the contribution of sulphated steroids to the regulation of decidualisation and expand our understanding of intracrine regulation of the endometrium during the establishment of pregnancy.

## Supplementary Material

Supporting Figure 1

Supporting Table 1

Supporting Table 2

Supporting Table 3

## Declaration of interest

The authors declare that there is no conflict of interest that could be perceived as prejudicing the impartiality of this review.

## Funding

Studies were supported by MRC Programme Grant G1100356/1 (P T K S); MRC grant MR/J003611/1 (to H O D C).

## Author contribution statement

Experimental design: D A G, P T K S, experimental procedures: D A G, P A F, I S, F C, O K, manuscript preparation: D A G, I S, P T K S.

## References

[bib1] Bombail V Gibson DA Collins F MacPherson S Critchley HO Saunders PT 2010 A role for the orphan nuclear receptor estrogen-related receptor alpha in endometrial stromal cell decidualization and expression of genes implicated in energy metabolism. Journal of Clinical Endocrinology and Metabolism 95 E224–E228. (10.1210/jc.2010-0154) 20668045 PMC3050102

[bib2] Colette S Defrere S Lousse JC Van Langendonckt A Gotteland JP Loumaye E Donnez J 2011 Inhibition of steroid sulfatase decreases endometriosis in an in vivo murine model. Human Reproduction 26 1362–1370. (10.1093/humrep/der079) 21441545

[bib4] Das A Mantena SR Kannan A Evans DB Bagchi MK Bagchi IC 2009 De novo synthesis of estrogen in pregnant uterus is critical for stromal decidualization and angiogenesis. PNAS 106 12542–12547. (10.1073/pnas.0901647106) 19620711 PMC2718343

[bib3] Das A Li Q Laws MJ Kaya H Bagchi MK Bagchi IC 2012 Estrogen-induced expression of Fos-related antigen 1 (FRA-1) regulates uterine stromal differentiation and remodeling. Journal of Biological Chemistry 287 19622–19630. (10.1074/jbc.M111.297663) 22514284 PMC3365997

[bib5] Dassen H Punyadeera C Kamps R Delvoux B Van Langendonckt A Donnez J Husen B Thole H Dunselman G Groothuis P 2007 Estrogen metabolizing enzymes in endometrium and endometriosis. Human Reproduction 22 3148–3158. (10.1093/humrep/dem310) 17921479

[bib6] Day JM Purohit A Tutill HJ Foster PA Woo LW Potter BV Reed MJ 2009 The development of steroid sulfatase inhibitors for hormone-dependent cancer therapy. Annals of the New York Academy of Sciences 1155 80–87. (10.1111/j.1749-6632.2008.03677.x) 19250195

[bib7] Falany JL Falany CN 1996 Expression of cytosolic sulfotransferases in normal mammary epithelial cells and breast cancer cell lines. Cancer Research 56 1551–1555. 8603401

[bib8] Foster PA Woo LW Potter BV Reed MJ Purohit A 2008 The use of steroid sulfatase inhibitors as a novel therapeutic strategy against hormone-dependent endometrial cancer. Endocrinology 149 4035–4042. (10.1210/en.2008-0223) 18450955 PMC2488239

[bib9] Fournier MA Poirier D 2009 Estrogen formation in endometrial and cervix cancer cell lines: involvement of aromatase, steroid sulfatase and 17beta-hydroxysteroid dehydrogenases (types 1, 5, 7 and 12). Molecular and Cellular Endocrinology 301 142–145. (10.1016/j.mce.2008.08.027) 18817841

[bib10] Gamage NU Tsvetanov S Duggleby RG McManus ME Martin JL 2005 The structure of human SULT1A1 crystallized with estradiol. An insight into active site plasticity and substrate inhibition with multi-ring substrates. Journal of Biological Chemistry 280 41482–41486. (10.1074/jbc.M508289200) 16221673

[bib11] Gellersen B Brosens JJ 2014 Cyclic decidualization of the human endometrium in reproductive health and failure. Endocrine Reviews 35 851–905. (10.1210/er.2014-1045) 25141152

[bib13] Gibson DA McInnes KJ Critchley HO Saunders PT 2013 Endometrial intracrinology – generation of an estrogen-dominated microenvironment in the secretory phase of women. Journal of Clinical Endocrinology and Metabolism 98 E1802–E1806. (10.1210/jc.2013-2140) 24014622

[bib12] Gibson DA Greaves E Critchley HO Saunders PT 2015 Estrogen-dependent regulation of human uterine natural killer cells promotes vascular remodelling via secretion of CCL2. Human Reproduction 30 1290–1301. (10.1093/humrep/dev067) 25820695 PMC4498222

[bib14] Gibson DA Simitsidellis I Cousins FL Critchley HO Saunders PT 2016a Intracrine androgens enhance decidualization and modulate expression of human endometrial receptivity genes. Scientitifc Reports 6 19970 (10.1038/srep19970) PMC4730211 26817618

[bib16] Gibson DA Simitsidellis I Saunders PT 2016b Regulation of androgen action during establishment of pregnancy. Journal of Molecular Endocrinology 57 R35–R47. (10.1530/JME-16-0027) 27067639

[bib15] Gibson DA Simitsidellis I Kelepouri O Critchley HOD Saunders PTK 2018 Dehydroepiandrosterone enhances decidualization in women of advanced reproductive age. Fertility and Sterility 109 728.e722–734.e722. (10.1016/j.fertnstert.2017.12.024) 29397924 PMC5908781

[bib17] Huhtinen K Desai R Stahle M Salminen A Handelsman DJ Perheentupa A Poutanen M 2012 Endometrial and endometriotic concentrations of estrone and estradiol are determined by local metabolism rather than circulating levels. Journal of Clinical Endocrinology and Metabolism 97 4228–4235. (10.1210/jc.2012-1154) 22969138 PMC3485603

[bib18] Koot YE van Hooff SR Boomsma CM van Leenen D Groot Koerkamp MJ Goddijn M Eijkemans MJ Fauser BC Holstege FC Macklon NS 2016 An endometrial gene expression signature accurately predicts recurrent implantation failure after IVF. Scientific Reports 6 19411 (10.1038/srep19411) 26797113 PMC4726345

[bib19] Liu HC Tseng L 1979 Estradiol metabolism in isolated human endometrial epithelial glands and stromal cells. Endocrinology 104 1674–1681. (10.1210/endo-104-6-1674) 446388

[bib20] Ma WG Song H Das SK Paria BC Dey SK 2003 Estrogen is a critical determinant that specifies the duration of the window of uterine receptivity for implantation. PNAS 100 2963–2968. (10.1073/pnas.0530162100) 12601161 PMC151449

[bib21] Miki Y Nakata T Suzuki T Darnel AD Moriya T Kaneko C Hidaka K Shiotsu Y Kusaka H Sasano H 2002 Systemic distribution of steroid sulfatase and estrogen sulfotransferase in human adult and fetal tissues. Journal of Clinical Endocrinology and Metabolism 87 5760–5768. (10.1210/jc.2002-020670) 12466383

[bib22] Mueller JW Gilligan LC Idkowiak J Arlt W Foster PA 2015 The regulation of steroid action by sulfation and desulfation. Endocrine Reviews 36 526–563. (10.1210/er.2015-1036) 26213785 PMC4591525

[bib23] Nishimura M Naito S 2005 Tissue-specific mRNA expression profiles of human ATP-binding cassette and solute carrier transporter superfamilies. Drug Metabolism and Pharmacokinetics 20 452–477. (10.2133/dmpk.20.452) 16415531

[bib24] Pellicer A Valbuena D Cano F Remohi J Simon C 1996 Lower implantation rates in high responders: evidence for an altered endocrine milieu during the preimplantation period. Fertility and Sterility 65 1190–1195. (10.1016/S0015-0282(16)58337-X) 8641496

[bib25] Piccinato CA Neme RM Torres N Sanches LR Derogis P Brudniewski HF Rosa ESJC Ferriani RA 2016 Effects of steroid hormone on estrogen sulfotransferase and on steroid sulfatase expression in endometriosis tissue and stromal cells. Journal of Steroid Biochemistry and Molecular Biology 158 117–126. (10.1016/j.jsbmb.2015.12.025) 26723541

[bib26] Purohit A Froome VA Wang DY Potter BV Reed MJ 1997 Measurement of oestrone sulphatase activity in white blood cells to monitor in vivo inhibition of steroid sulphatase activity by oestrone-3-O-sulphamate. Journal of Steroid Biochemistry and Molecular Biology 62 45–51. (10.1016/S0960-0760(97)00018-6) 9366497

[bib27] Purohit A Woo LW Potter BV 2011 Steroid sulfatase: a pivotal player in estrogen synthesis and metabolism. Molecular and Cellular Endocrinology 340 154–160. (10.1016/j.mce.2011.06.012) 21693170

[bib28] Rizner TL Thalhammer T Ozvegy-Laczka C 2017 The importance of steroid uptake and intracrine action in endometrial and ovarian cancers. Frontiers in Pharmacology 8 346 (10.3389/fphar.2017.00346) 28674494 PMC5474471

[bib29] Rubin GL Harrold AJ Mills JA Falany CN Coughtrie MW 1999 Regulation of sulphotransferase expression in the endometrium during the menstrual cycle, by oral contraceptives and during early pregnancy. Molecular Human Reproduction 5 995–1002. (10.1093/molehr/5.11.995) 10541560

[bib30] Ruiz-Alonso M Blesa D Diaz-Gimeno P Gomez E Fernandez-Sanchez M Carranza F Carrera J Vilella F Pellicer A Simon C 2013 The endometrial receptivity array for diagnosis and personalized embryo transfer as a treatment for patients with repeated implantation failure. Fertility and Sterility 100 818–824. (10.1016/j.fertnstert.2013.05.004) 23756099

[bib31] Schroder E Gebel L Eremeev AA Morgner J Grum D Knauer SK Bayer P Mueller JW 2012 Human PAPS synthase isoforms are dynamically regulated enzymes with access to nucleus and cytoplasm. PLoS ONE 7 e29559 (10.1371/journal.pone.0029559) 22242175 PMC3252339

[bib32] Simpson ER Mahendroo MS Means GD Kilgore MW Hinshelwood MM Graham-Lorence S Amarneh B Ito Y Fisher CR Michael MD, ***et al*** 1994 Aromatase cytochrome P450, the enzyme responsible for estrogen biosynthesis. Endocrine Reviews 15 342–355. (10.1210/edrv-15-3-342) 8076586

[bib33] Sinreih M Knific T Anko M Hevir N Vouk K Jerin A Frkovic Grazio S Rizner TL 2017 The significance of the sulfatase pathway for local estrogen formation in endometrial cancer. Frontiers in Pharmacology 8 368 (10.3389/fphar.2017.00368) 28690541 PMC5481366

[bib34] Smuc T Rupreht R Sinkovec J Adamski J Rizner TL 2006 Expression analysis of estrogen-metabolizing enzymes in human endometrial cancer. Molecular and Cellular Endocrinology 248 114–117. (10.1016/j.mce.2005.10.013) 16337331

[bib35] Suzuki T Miki Y Nakata T Shiotsu Y Akinaga S Inoue K Ishida T Kimura M Moriya T Sasano H 2003 Steroid sulfatase and estrogen sulfotransferase in normal human tissue and breast carcinoma. Journal of Steroid Biochemistry and Molecular Biology 86 449–454. (10.1016/S0960-0760(03)00356-X) 14623543

[bib36] Utsunomiya H Ito K Suzuki T Kitamura T Kaneko C Nakata T Niikura H Okamura K Yaegashi N Sasano H 2004 Steroid sulfatase and estrogen sulfotransferase in human endometrial carcinoma. Clinical Cancer Research 10 5850–5856. (10.1158/1078-0432.CCR-04-0040) 15355916

