# Supplementary figures and images for: SULFATION PATHWAYS: A role for steroid sulphatase in intracrine regulation of endometrial decidualisation
Source: J Mol Endocrinol. 2018 May 2;61(2):M57–65. doi: 10.1530/JME-18-0037 (PMC6055542; doi:10.1530/JME-18-0037)

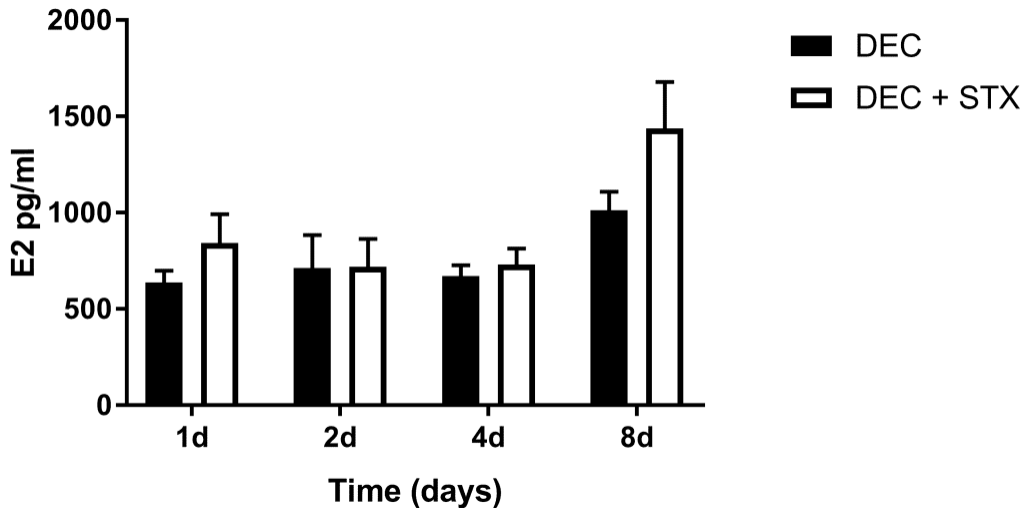

Supplement: Supporting Figure 1 [file jme-60-M57-s001.pdf]
