# Supplementary material for: SULFATION PATHWAYS: A role for steroid sulphatase in intracrine regulation of endometrial decidualisation
Source: J Mol Endocrinol. 2018 May 2;61(2):M57–65. doi: 10.1530/JME-18-0037 (PMC6055542; doi:10.1530/JME-18-0037)
Supplement: Supporting Table 1 [file jme-60-M57-t001.pdf]

Supplementary Table 1 - Primers and probes for qRTPCR.

| GOI | 5' to 3' | 3' to 5' | UPL probe |
|---|---|---|---|
| ***STS*** | cggaagtaatgggatctataaagg | aacgaaggatgcctggaac | 12 |
| ***SULT1E1*** | tcattttgggaaaaggattgtaa | ccagccaccattagaaagaaa | 5 |
| ***SULT1A1*** | cccacgactcctgaagacac | gcgggcaacatagaccac | 32 |
| ***SULT2A1*** | tgagagaggagaaaaacttcctgt | tctttcccaggaattgacaga | 17 |
| ***SULT2B1 v1 (SULT2B1a)*** | aacttcctatttatcacctacgagga | ccaggaacccacagatgc | 18 |
| ***SLCO1A2*** | ggggcatgcaggatatatga | tggaacaaagcttgatcctctt | 77 |
| ***SLCO2B1*** | ataccgctacgacaacacca | tgagcagttgccattggag | 57 |
| ***SLCO1B3*** | gcatagccctgaagtgttgtc | ctgatgcttggtttgaaatgat | 10 |
| ***SLCO1B1*** | ttcaagtggcaataaaaagccta | cacccaaatgggctgagtaa | 54 |
| ***ABCC1*** | aaggcctattaccccagcat | atgcagttgcccacacact | 83 |
| ***ABCC4*** | gaagcgcctggaatctacaa | agagcccctggagagaagat | 47 |
| ***PAPSS1*** | tcccatgatgtatgctggac | aaagttggctcctgcaacc | 59 |
| ***PAPSS2*** | tgggggacaacatgtacaaa | aaccagccagtccccact | 9 |
