# Supplementary material for: SULFATION PATHWAYS: A role for steroid sulphatase in intracrine regulation of endometrial decidualisation
Source: J Mol Endocrinol. 2018 May 2;61(2):M57–65. doi: 10.1530/JME-18-0037 (PMC6055542; doi:10.1530/JME-18-0037)
Supplement: Supporting Table 2 [file jme-60-M57-t002.pdf]

Supplementary table 2 - Cross-reactivity of estrone ELISA

**STEROID**	**% CROSS-REACTIVITY**
Estrone	100
Estrone-3-Sulfate	4.9
17β-Estradiol	2.2
Estrone-3-Glucuronide	1.2
17β-Estradiol-3-Glucuronide	0.14
Androstenedione, Cholesterol, Corticosterone, Cortisol, Cortisone, DHEAS, Diethylstilbesterone, Estriol, Estradiol-Sulfate, Progesterone, 17-OH Progesterone, Testosterone.	0.1
