# Supplementary material for: SULFATION PATHWAYS: A role for steroid sulphatase in intracrine regulation of endometrial decidualisation
Source: J Mol Endocrinol. 2018 May 2;61(2):M57–65. doi: 10.1530/JME-18-0037 (PMC6055542; doi:10.1530/JME-18-0037)
Supplement: Supporting Table 3 [file jme-60-M57-t003.pdf]

Supplementary table 3 - Cross-reactivity of estradiol ELISA

**STEROID**	**% CROSS-REACTIVITY**
Estradiol	100
Estrone	<2
Estriol	<0.15
Testosterone	<0.01
DHEA	<0.01
Androstenedione	0
